# CURA—An Ethics Support Instrument for Nurses in Palliative Care. Feasibility and First Perceived Outcomes

**DOI:** 10.1007/s10730-021-09456-6

**Published:** 2021-12-09

**Authors:** Malene Vera van Schaik, H. Roeline Pasman, Guy Widdershoven, Bert Molewijk, Suzanne Metselaar

**Affiliations:** 1 Amsterdam UMC, Location VU Medical Centre, Amsterdam, The Netherlands; 2 Centre for Medical Ethics, University of Oslo, Oslo, Norway; 3 Department of Ethics, Law and Humanities, Amsterdam UMC Location VUmc, De Boelelaan 1089a, 1081 HV Amsterdam, The Netherlands

**Keywords:** Clinical ethics support, Nurses, Palliative care, Ethics, Moral resilience

## Abstract

Evaluating the feasibility and first perceived outcomes of a newly developed clinical ethics support instrument called CURA. This instrument is tailored to the needs of nurses that provide palliative care and is intended to foster both moral competences and moral resilience. This study is a descriptive cross-sectional evaluation study. Respondents consisted of nurses and nurse assistants (n = 97) following a continuing education program (course participants) and colleagues of these course participants (n = 124). Two questionnaires with five-point Likert scales were used. The feasibility questionnaire was given to all respondents, the perceived outcomes questionnaire only to the course participants. Data collection took place over a period of six months. Respondents were predominantly positive on most items of the feasibility questionnaire. The steps of CURA are clearly described (84% of course participants agreed or strongly agreed, 94% of colleagues) and easy to apply (78–87%). The perceived outcomes showed that CURA helped respondents to reflect on moral challenges (71% (strongly) agreed), in perspective taking (67%), with being aware of moral challenges (63%) and in dealing with moral distress (54%). Respondents did experience organizational barriers: only half of the respondents (strongly) agreed that they could easily find time for using CURA. CURA is a feasible instrument for nurses and nurse assistants providing palliative care. However, reported difficulties in organizing and making time for reflections with CURA indicate organizational preconditions ought to be met in order to implement CURA in daily practice. Furthermore, these results indicate that CURA helps to build moral competences and fosters moral resilience.

## Introduction

Palliative care is seen as a “deeply ethical practice”, which may have a profound impact on caregivers (Leget, 2018). Correspondingly, caregivers providing palliative care are known to frequently experience moral challenges in their work (Brazil et al., 2009; Frey et al., 2018; Hermsen & Ten Have, 2001, Oberle & Hughes, 2001). Moral challenges arise from situations in which people can no longer rely on their moral routines, i.e., when there is an internal conflict between the norms and values that (implicitly) guide them in their work (Parker, 2015), which leads to moral doubt. Alternatively, moral challenges are experienced when those involved in the situation (for instance the nurse and the physician) do not agree on what is “good care” in the situation at hand. Finally, a situation could be experienced as a moral challenge because what is seen as “good care” is not in line with common practice, laws or guidelines.

Caregivers working in palliative care may, for instance, experience moral challenges in situations where professionals are confronted with continuation of curative treatment while they feel palliative care might be more appropriate; in relation to the role of informal caregivers and/or when organizational guidelines are in conflict with the patient’s last wishes (Brazil et al., 2009; Maffoni et al., 2019).

These moral challenges may lead to feelings of “moral distress”, i.e., a negative psychological event that is causally related to the experience of a moral challenge (Morley et al., 2019). Building moral competences, such as recognizing and reflecting on moral challenges, so as to come to well-considered actions, helps in dealing with moral distress (Bodenheimer & Sinsky, 2014; Frey et al., 2018; McHugh et al., 2011; Rushton, 2013).

Furthermore, being able to deal well with moral challenges is beneficial for the quality of (palliative) care itself (Abma et al., 2009). Clinical Ethics Support (CES) seeks to support caregivers in doing so and to foster their moral competences (Haan et al., 2018; Metselaar et al., 2015; Van der Dam et al., 2013). Various CES instruments are used in order to promote methodically structured reflection, such as moral case deliberation (MCD), ethics rounds or Ethical Reflection Groups (Grönlund et al., 2016; Kälvemark Sporrong et al., 2007; Molewijk et al., 2008; Rasoal et al., 2016; Silén et al., 2016; Stolper et al., 2016). Studies show promising results with regard to the effects of CES instruments on the moral competences of caregivers (De Snoo-Trimp et al., 2019; Janssens et al., 2015; Kälvemark Sporrong et al., 2007; Lillemoen & Pedersen, 2015; Svantesson et al., 2019; Söderhamn et al., 2014).

In our study, we developed a CES instrument especially designed for caregivers in palliative care—nurses in particular. The participants in our study as well as those in previous studies conducted in a similar care setting (Dutch elderly care) (Hartman et al., 2019) indicated that existing CES instruments have limitations in practice, for instance related to their complexity or because they are too time-consuming, and therefore difficult to integrate in daily practice.

Taking the above as our point of departure, we developed a four-step instrument called CURA for and in close collaboration with caregivers in palliative care. CURA presents a method for ethical reflection that is in line with the principles that underlie MCD, i.e., that of dialogical and hermeneutic ethics (Widdershoven & Molewijk, 2010; Widdershoven & Metselaar, 2012), but it is also specifically tailored to the needs and wishes of caregivers in palliative care and seeks to mitigate the aforementioned limitations of existing CES instruments.

However, the question remains whether CURA is indeed feasible in practice, taking into account that palliative care is practiced in many different health care settings, and whether it indeed improves the ability of caregivers—especially nurses—to deal with morally challenging situations. Therefore, the aim of the study presented here is to investigate this feasibility as well as the perceived first outcomes of using CURA.

### Background: Development Study

CURA was developed in a two-year study that was part of a large national research program in order to improve the quality of palliative care in The Netherlands. Central to the participatory design of our study was a “community of practice” (CoP); a group of people who share a concern or a passion for something they do and that develop new or improve existing practices or interventions together (Wenger, 2011). Our CoP mainly consisted of health care professionals, implementation experts, patient representatives, and education professionals. All were involved in palliative care.

At the start of our study, the CoP members unanimously expressed the need for a CES instrument with low complexity (structure and language), which would be useable without elaborate training, and which would attribute a central place to the needs, wishes and values of all stakeholders (especially the patient) in a situation that is experienced as morally troublesome. Also, they indicated that the instrument should be relatively time-efficient, as this would make it easier to integrate CES into daily practice.

Taking into account these criteria, we came to a first draft, taking existing CES instruments within the dialogical and hermeneutic tradition in CES as our point of departure (Hartman et al., 2019; Stolper et al., 2016) because of its focus on fostering moral learning processes of caregivers (Metselaar et al., 2015). We tested multiple drafts of the instrument in several try-out groups consisting of nurses and nurse assistants. The findings from these try-out groups as well as the feedback of our CoP during work sessions helped us to further refine the concept-instrument in iterative cycles. This resulted in CURA (provided in the Appendix).

### CURA

CURA is designed to provide a low-threshold structure for methodical reflection on moral challenges in palliative care. It can be used independently after a relatively short introduction. CURA can be used both individually and in (smaller) groups. A reflection with CURA can be done in a relatively short time frame (30–45 minutes).

CURA is an acronym for the four steps of the instrument: (1) Concentrate, (2) Unrush, (3) Reflect, and (4) Act. In the first step, Concentrate, the users focus on describing the situation that is experienced as morally troublesome and on articulating the moral doubts that arise from that situation. If CURA is used together, participants of the joint reflection may ask each other questions in order to elucidate the situation and doubts.

The aim of the second step, Unrush, is to become aware of and explore one’s initial response to the situation. This response may involve emotions, judgments, and even a physical reaction to the situation. When all participants have expressed and explored their initial responses, they are instructed to postpone or “park” them in order to be able to venture into the stakeholders’ perspectives with an open mind.

The third step, Reflect, focuses on deepening one’s understanding of the case by exploring what is or what could be important to those involved in the situation, starting with the patient. This includes self-reflection on one’s own moral perspective on the situation. Relevant guidelines and protocols are also taken into account in this step.

The fourth step is Act. First, users balance and weigh everything that has come up as important in the reflection, and assess what is most important to them, i.e., what should be prioritized in taking action. Subsequently, they consider how their chosen course of action relates to what they deem valuable in providing palliative care.

CURA differs from other four-step approaches in CES such as the Four Boxes approach (Jonsen et al., 2010) or the four principles approach (Beauchamp & Childress, 2001). Primarily, because it does not prescribe which principles or values need to be taken into consideration when deliberating on an ethical issue. Rather, in line with a dialogical and hermeneutic approach to ethics, the four steps of CURA seek to foster a joint moral learning process in which what is of moral importance comes up from exploring the situation at hand and the perspectives of those involved (Metselaar et al., 2015). The explicit attention for (the moral value of) emotions is another distinctive feature of CURA. Reflecting on emotions can deepen our understanding of what is of moral value to us in a specific situation (Nussbaum, 1990; Molewijk et al., 2011). Furthermore, sharing our emotions in a non-judgmental way may help with processing a morally difficult situation, which is considered a useful strategy to strengthen one's moral resilience, i.e., the “capacity to sustain, restore or deepen their integrity in response to moral complexity, confusion, distress or setbacks” (Rushton et al., 2016).

Compared to other CES instruments within the dialogical and hermeneutic tradition, CURA has a relatively simple structure. It is not necessary to engage an ethicist or trained facilitator to guide the process, whereas other forms of moral deliberation often need such a facilitator (Stolper et al., 2016) and are often more time-consuming (Hartman et al., 2019).

## Method

### Design

The study was designed as a descriptive cross-sectional evaluation study of the feasibility and perceived first outcomes of CURA, using questionnaires based on validated instruments for feasibility and perceived outcomes.

### Respondents

We included two groups of respondents. The first group consisted of nurses who were course participants (n = 97). The course participants were part-time students following a continuing education program while they were already working as registered nurses (RN), licensed practical nurses (LPN) or certified nursing assistants (CNA). Their training focused either on a specialization in a specific patient population (Oncology, Cardiac Care, Neurology or Geriatrics), or they were enrolled in art-time vocational training to get a degree as a LPN.

The second group consisted of the colleagues of the course participants (n = 124). Course participants asked up to three colleagues to use CURA with them. All participants worked in different organizations and settings.

Course participants were asked to fill in both questionnaires, i.e., the feasibility and perceived outcomes questionnaire, if they had attended all meetings on CURA and used CURA together with their colleagues. Colleagues were asked to only fill in the feasibility questionnaire if they had used CURA together with one of the course participants.

### Introduction and Use of CURA

The course participants received an introduction in CURA as part of their ethics course. This ethics course consisted of either two or three lessons. In these lessons, they practiced CURA under the supervision of the teacher/researcher and received a manual that provided instructions and general information on the goals and use of CURA in daily practice. Then they used CURA with their colleagues in their own work setting, either during working hours or after their shift. Subsequently, the course participants asked at least one of their colleagues to complete a questionnaire on the feasibility of CURA.

### Data Collection

Two questionnaires were used. Data collection took place from January 2019 to June 2019.

#### Feasibility Questionnaire

This questionnaire is based on the validated measurement instrument MIDI (‘the Measurement Instrument for Determinants of Innovation’) (Fleuren et al., 2014). This instrument describes relevant determinants that may affect implementation and feasibility of interventions in health care and gives suggestions on how each determinant should be measured. Researchers can choose which determinants should be included in their study. For this study, 7 of 29 determinants were selected. Selection was based on whether respondents could form a well-considered opinion on the determinant after using CURA for a short amount of time. We predominantly chose determinants associated with the innovation itself (CURA) and the user, and to a less extent determinants associated with the organization and socio-political context. The course participants received three additional questions concerning the introduction and two questions regarding the use of CURA individually and with colleagues. The seven selected determinants and items can be found in Table 1. Items were measured using a 5-point Likert scale. All respondents filled in the feasibility questionnaire.

**Table 1:** Determinants and items feasibility questionnaire

Determinant	Items in feasibility questionnaire
Procedural clarity	The steps of CURA are clearly described The steps of CURA follow each other in logical order
Complexity	The steps of CURA are easy to go through The language used in CURA is understandable CURA is easy to use by myself^a^ CURA is easy to use together with colleagues^a^
Compatibility	CURA is easy to use in daily practice
Descriptive norm	I think my colleagues (nurses and nurse assistants) are open minded towards using CURA
Self-efficacy	The next time I encounter a morally difficult situation, I would use CURA
Time	I could easily make time for using CURA The time investment is in proportion to what it provides in terms of insight and reflection
Information	I have received ample introduction (classes/explanation) to use CURA independently with colleagues^a^ I have had enough time to practice with CURA during the classes on CURA^a^ The manual is supportive when using CURA^a^

^a^These items were only used with the course participants (n = 97)

#### Perceived Outcomes Questionnaire

The perceived outcomes questionnaire was based on the partially validated Euro-MCD instrument measuring the experienced outcomes of moral case deliberation (MCD) (Svantesson et al., 2014). We chose the Euro-MCD because of the similarities between MCD and CURA: both instruments are rooted in hermeneutic and dialogical ethics, regarding concrete experience as the source of moral knowledge (Widdershoven et al., 2009) and are aimed at developing moral competences (Metselaar et al., 2015). The Euro-MCD consists of 26 possible outcomes of MCD (Svantesson et al., 2014).

We selected 11 items for our questionnaire based on their relevance to the intended aims of CURA (fostering moral competences, such as: creating awareness of moral challenges, perspective taking, moral reflexivity, well-considered action taking and decrease of moral distress). The items were reformulated and specified to the use of CURA. We used the term “morally difficult situations” to emphasize CURA is intended to use for concrete experiences in practice. Items were measured using a 5-point Likert scale.

The perceived outcomes questionnaire was only given to the course participants who had received an introduction in using CURA and used CURA at least two times. The colleagues of course participants had only used CURA once and did not receive any introduction by the researchers. We presumed they did not have enough experience to answer questions on the perceived outcomes (Table 2).

**Table 2:** Items in perceived outcomes study

1. CURA helps me to reflect on morally difficult situations
2. CURA helps me to deal with stress caused by morally difficult situations
3. CURA is important for my daily work practice
4. CURA helps me to express my doubts about morally difficult situations
5. CURA helps me to take the perspective of other stakeholders (such as the patient, other professionals, family etc.)
6. CURA helps me to become more aware of morally difficult situations
7. CURA helps me to take concrete actions in morally difficult situations
8. CURA helps me to become more aware of my emotions with regard to morally difficult situations
9. CURA reinforces my self-confidence in dealing with morally difficult situations
10. CURA helps me understand what it means to be a good professional
11. CURA helps me to clarify a morally difficult situation

### Data Analysis

Descriptive statistics were used to investigate the characteristics and responses on feasibility and perceived outcomes. Missing data was random and no item exceeded more than 3 missing responses per item (max. 2.4%) on the items of the feasibility questionnaire. There was no missing data on the perceived outcomes questionnaire. The answer categories “strongly disagree” and “disagree” were merged into a single category because of small frequencies. In order to give detailed insight in the dispersion of the results, we chose to present the percentage of each answer category, rather than merely presenting means and median numbers per item. We did not use in-between group comparisons because our groups were too small for comparison. Analyzes were performed using SPSS version 26.

### Ethical Considerations

Ethical approval was granted by Institutional Review board (IRB) of Amsterdam UMC, location VUmc. This study was considered not subject to the Medical Research Involving Human Subject Act (WMO). Both questionnaires stated clearly that results would be used for scientific purposes, and that respondents gave informed consent by filling in the questionnaire. Researchers emphasized to course participants it was not obligatory to fill in the questionnaires. However, all students that were eligible filled in the questionnaires. The questionnaires were anonymous and no form of personal identification was asked: we used codes to identify subgroups.

## Results

### Characteristics

Demographic and professional characteristics of respondents are summarized in Table 3. A majority of the course participants and colleagues of course participants were female (resp. 93%/85%).^1^ Most were Licensed Practical Nurses (46%/34%), followed by Registered Nurses (39%/26%) and worked in hospital setting (72%/67%). A small portion worked in home care (4%/6%).

**Table 3:** Respondent characteristics

	Course participants (n/%) (n = 97)	Colleagues of course participants (n/%) (n = 124)
Female gender^a^	90 (93%)	105 (85%)
Years of experience	Mean: 8 years	Mean: 10 years
0–5	53 (55%)	51 (41%)
6–10	21 (22%)	33 (27%)
11–20	11 (11%)	14 (11%)
> 20	7 (7%)	18 (15%)
Profession		
Certified nurse assistant	9 (9%)	21 (17%)
Licensed nurse practitioner	45 (46%)	42 (34%)
Registered nurse	38 (39%)	32 (26%)
Other	2 (2%)	15 (12%)
Setting		
Home care	4 (4%)	7 (6%)
Nursing home/rehabilitation center	21 (22%)	29 (23%)
Hospital	70 (72%)	85 (67%)
Familiar with ethics support		
Yes, frequently used	14 (14%)	28 (23%)
Yes, sporadically	40 (41%)	55 (44%)
No, not familiar	37 (38%)	39 (32%)
Number of times CURA used		
1–2 times	88 (91%)	NA
3 and more	7 (7%)	
Duration of CURA		
0–10 min	4 (4%)	NA
11–20 min	36 (37%)	
21–30	39 (40%)	
31–40	6 (6%)	
> 40	10 (10%)	

^a^Missing scores were between 2 and 6% on all questions, except for “profession” of colleagues: 11% was missing

Most respondents had less than 5 years of experience in their current profession (55%/41%). We found that most respondents were to some extent familiar with other forms of clinical ethics support and used it sporadically or had used it (41%/44%). 38% of course participants and 32% of the colleagues were not familiar with CES. A smaller percentage used it frequently (14%/23%).

The course participants were also asked how often they had used CURA (including its introduction) and the duration of using CURA the last time they used it. The mean duration of CURA was 28 minutes, ranging from 5 to 60 minutes with one outlier of 120 minutes. Most respondents completed CURA within 30 minutes or less (81%). Most respondents had used CURA two times when filling in the questionnaires (91%).

### Feasibility of CURA

**Fig. 1:**
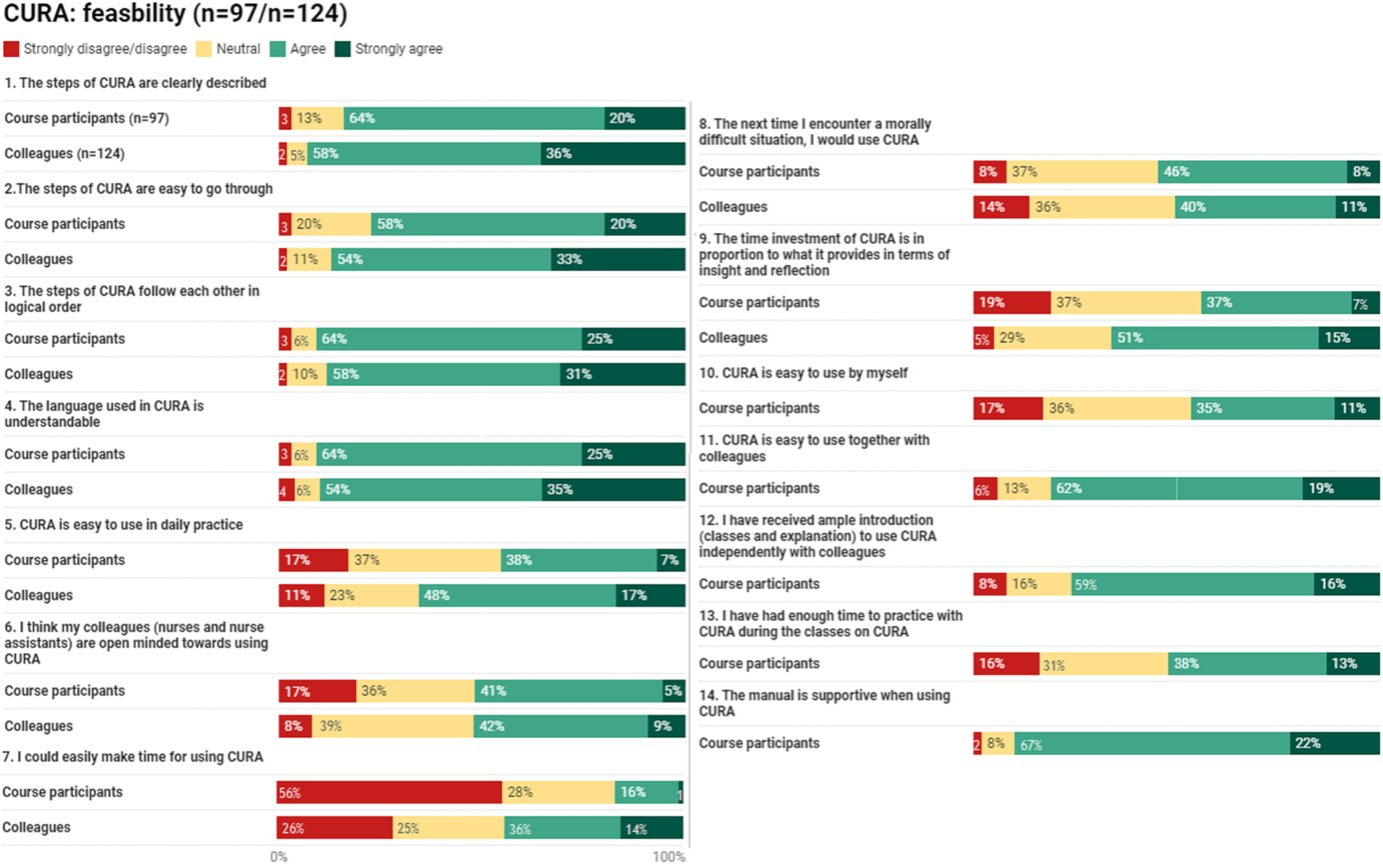
Feasibility questionnaire. Missing data: max. 2,4% per item

The results of the feasibility questionnaire are presented in Fig. 1. For most items the mode score was “agree”. On most items, the colleagues of course participants gave higher scores than the course participants. Items measuring the determinants “procedural clarity” and “complexity” got the highest scores. On the item *“the steps are clearly described”*, measuring “procedural clarity”, 84% of course participants and 94% of the colleagues agreed or strongly agreed. Other items measuring these determinants were *“the language used by CURA is understandable”* (89%/89%) and *“the steps of CURA follow each other in a logical order”* (89%/89%). We used two items to measure the determinant “time”. The item *“I could easily make time for using CURA”* got the lowest scores: 56% of the course participants (strongly) disagreed. Among colleagues, this percentage was 26%. On the other it*em “the time investment is in proportion to what CURA provides in terms of insight and reflection”*, 44%/66% (strongly) agreed. Colleagues were significantly more positive on the items measuring “time”.

On the item “*I think my colleagues are open minded towards using CURA”* 46%/51% (strongly) agreed. On the item “*the next time I encounter a morally difficult situation, I would use CURA”*, measuring self-efficacy, 54%/51% (strongly) agreed. A small group (8%/14% (strongly) disagreed.

On the item *“CURA is easy to use in daily practice”*, measuring the determinant “compatibility”, 45%/65%, (strongly) agreed.

The items on the introduction respondents received and the complexity of CURA when using the instrument either individually or in small group settings were only asked to the course participants (see Method section). On the item “*CURA is easy to use together with colleagues”*, 81% (strongly) agreed. 46% stated that CURA is easy to use by themselves. 36% were neutral with regard to using CURA alone.

Most respondents have had sufficient introduction to use CURA independently (75% (strongly) agreed). A substantive portion (strongly) disagreed or were neutral to the extent of time to practice with CURA (16% (strongly) disagree, 31% neutral). The manual was considered supportive: 89% (strongly) agreed.

### Perceived Outcomes of CURA

**Fig. 2:**
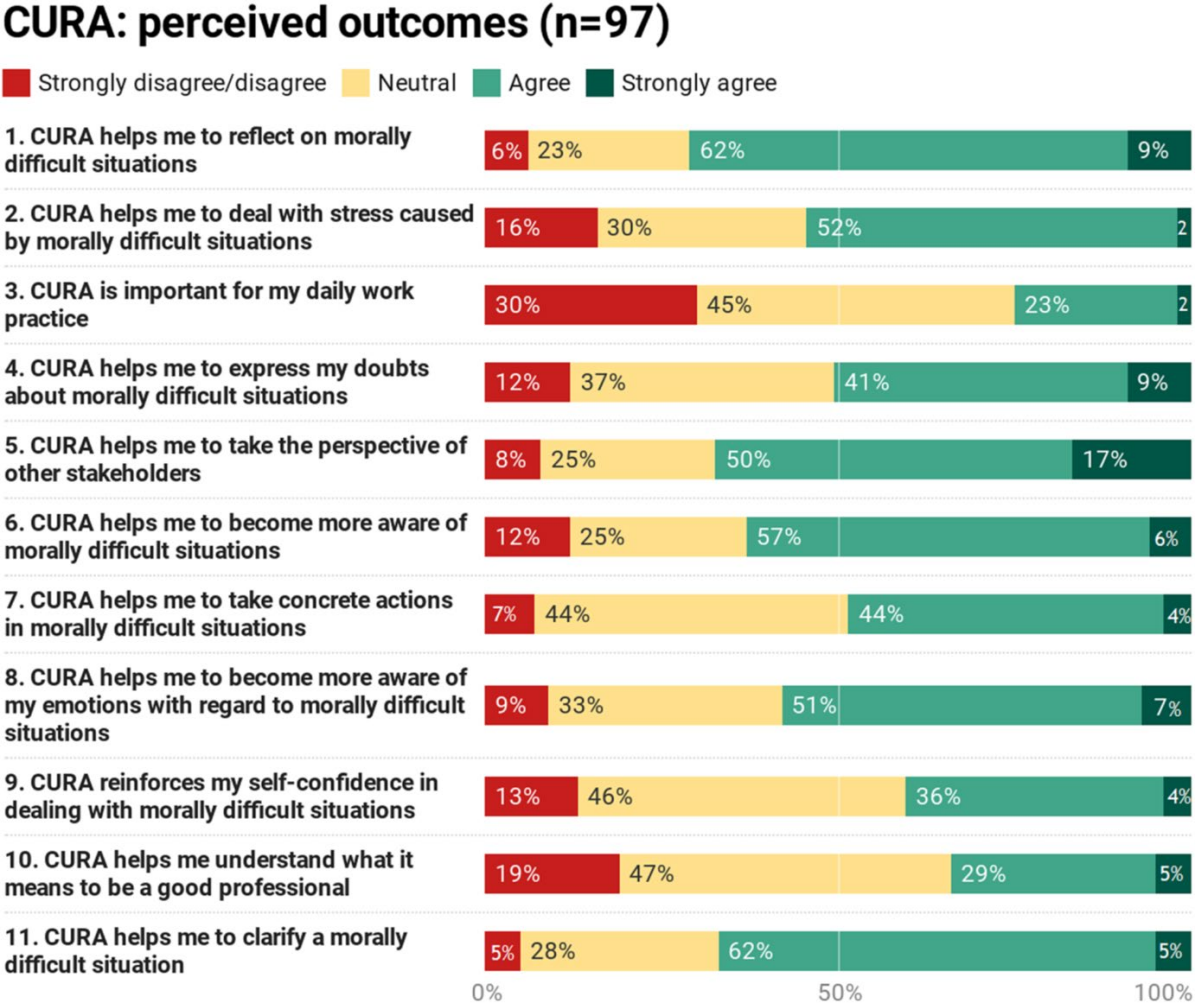
Perceived outcomes questionnaire. No missing data

The results of the perceived outcomes questionnaire are presented in Fig. 2. For most items the mode score was “agree”. Items with the highest scores were “*CURA helps me to reflect on morally difficult situations”* (71% (strongly) agreed); *“*CURA helps me to take the perspective of other stakeholders*”* (67%); *“CURA helps me to clarify a morally difficult situation*” (67%) and *“CURA helps me to become more aware of morally difficult situations”* (63%).

The items with highest scores on “(strongly) disagree” were *“CURA is important for my daily work practice”* (30% (strongly) disagree); *“CURA helps me understand what it means to be a good professional”* (19%); *“CURA helps me deal with distress caused by morally difficult situations”* (16%). However, the latter item also received a high score on (strongly) agree of 54%. On all these three items, the answer option “neutral” scored between 23 and 47%.

In specific, the items “*CURA helps me understand what it means to be a good professional” (*47% neutral) and *“CURA reinforces my self-confidence in dealing with morally difficult situations”* (46%) received the highest scores on this answer category.

There was no significant association between respondents who frequently used other forms of CES compared to respondents who were unfamiliar with other forms of CES.

## Discussion

### Feasibility

CURA is intended to be low-threshold in use, meaning it should be applicable in all care settings in which palliative care is provided, and can be used both individually and in small group settings in a relatively short time period, without extensive training or the guidance of a trained facilitator or ethicist. It took most respondents 25–30 minutes to use CURA, which is considerably shorter than other forms of CES, such as MCD (Haan et al., 2018; Hartman et al., 2019). This increases its applicability in practice.

The determinants measuring the instrument (CURA) itself, i.e., “procedural clarity” and “complexity” scored positively, meaning the instrument follows a logical order; the language is clear and understandable. Furthermore, the determinant “information”, measuring whether users received ample instruction and information to use CURA independently, scored high as well. However, a substantive number of respondents say that they will need more time to practice with CURA during the introduction session. We will take this into account in fine-tuning the introduction to CURA, as well as in the development of an envisioned training module for the implementation of CURA by “CURA-ambassadors”, i.e., people who can take the lead in introducing, initiating and organizing CURA in their organization.

Items that measured the determinants associated with the end-users and the organizational setting such as *“I could easily make time for CURA”*; *“CURA is easy to use in daily practice”* and “*I think my colleagues are open minded towards using CURA”* received lower scores. This is an indication that respondents experienced obstacles to using CURA, such as time constraints and application in daily practice. This finding is consistent with other research on implementing CES in healthcare organizations (Söderhamn et al., 2014; Haan et al., 2018) and other interventions in palliative care (Verberne et al., 2018). When confronted with time pressure, nurses prioritize direct patient care with immediate and visible impact (Vinckx et al., 2018).

While CURA is less time-consuming than other types of CES, our results show that it is still challenging for nurses to find enough time to use CURA. This raises the question whether we should find ways to develop an instrument which is even less time-consuming—if this is even possible and desirable. Rather, it might be more useful to focus on organizational and systemic issues when implementing CES. Studies have shown that moral distress is related to other factors, such as time pressure and consultation opportunities with colleagues (De Veer et al., 2013; Lamiani et al., 2017). Consequently, not only the type of CES itself but also the organizational preconditions are important aspects of successfully embedding CES in daily practice.

In the implementation research that is currently being conducted, we specifically pay attention to the inhibiting and promoting factors that influence the use and implementation of CURA in certain contexts. CURA might be deemed a feasible instrument based on its characteristics, however, other factors in the organizational setting (such as availability of time, a safe ethical work environment, commitment from all levels within the organization) are known to be relevant for successful implementation of new interventions in practice (Cummings et al., 2007; Damschroder et al, 2009; Fleuren et al., 2004; Kälvemark Sporrong et al., 2007; Oh & Gastmans, 2015; Rushton & Sharma, 2018;). Conducting evaluation research provides in-depth understanding of the feasibility of a new CES instrument (Schildmann et al., 2019) and may help to identify barriers in an early stage which provides opportunities for resolution (Campbell et al., 2007). Although CURA has advantages compared to other forms of CES, organizational and contextual preconditions require attention in the implementation process.

### Perceived Outcomes

A majority of the respondents in this study reported that CURA was helpful in developing moral competences. These findings are consistent with other studies on the impact of CES on moral competences (De Snoo- Trimp et al., 2019; Haan et al., 2018; Söderhamn et al., 2014; Tanner et al., 2014). In a study by De Snoo-Trimp et al. (2019), the perceived outcome “perspective taking” was scored as one of the highest items in their study, similar to the results of this study. Other studies found that CES strengthens moral competences, such as increased awareness of moral challenges (Söderhamn et al., 2014; Haan et al., 2018), being able to express one’s emotions (Molewijk et al., 2011) and to reflect on morally challenging situations (Silen et al., 2016; Söderhamn et al., 2014).

In palliative care, moral distress is a relevant phenomenon, specifically among nurses (Bender et al., 2019; Brazil et al., 2009; Kamal et al., 2016; Parola et al., 2017). Empirical studies relate CES to the reduction of moral distress among professionals (Tanner et al., 2014; Van der Dam et al., 2013). In our study, a small majority (strongly) agreed to the item measuring the extent to which CURA helps to manage distress caused by moral challenges. In the study by De Snoo-Trimp et al. (2019), this outcome was experienced the least compared to other positive effects by professionals attending ethics reflection sessions (with another method, i.e., Moral Case Deliberation). Several researchers stress the importance of appropriate ethics support to mitigate the detrimental effect of moral distress (Frey et al., 2018; Molewijk et al., 2011). As moral distress most likely cannot be eradicated (Rushton, 2017; 2018), Rushton proposes a shift from fighting moral distress towards cultivating moral resilience as a method to transform moral distress and to restore one’s moral resilience when it is imperiled (Rushton, 2018). Likewise, in the end, the aim of CURA is to cultivate nurses’ moral resilience. Further research is needed to investigate whether CURA indeed strengthens moral resilience.

Overall, the results were positive. Notably, when respondents did not agree or agree strongly with an item, they tended to choose the answer category “neutral”. Few items were disagreed with or disagreed with strongly. A possible explanation for this neutrality is that it is difficult for most respondents to assess the perceived outcomes on the basis of using CURA only a couple of times before completing the questionnaire. Therefore, respondents might have been reluctant to make strong claims about perceived outcomes or simply did not perceive any outcomes *yet*.

All in all, the results of this feasibility and perceived outcomes are promising but preliminary. They call for further research to (1) assess whether using CURA contributes to the moral competences of nurses in health care practice as well as in training when used repeatedly and over a longer period of time, and (2) to provide insights into how CURA can be effectively implemented in various palliative care settings as well as educational settings.

### Strengths and Limitations

One of the strengths of this study is that we included respondents who received an introduction in CURA as part of their obligatory ethics course during their vocational or post-graduate training. This limited drop-outs and selection bias. Furthermore, by letting course participants test CURA in their own work environment in diverse care contexts, we gained insight in the extent to which CURA is feasible in daily care practices.

However, asking the course participants to evaluate CURA in the context of their training (even though they were neither graded for this evaluation, nor was it an explicit assignment necessary to pass the course), might have encouraged them to give a more positive evaluation. Selection bias possibly occurred in the group of colleagues of course participants. Course participants presumably approached colleagues that have an open attitude towards new interventions and/or reflection and that were able to make time. On most items of the feasibility questionnaire, the colleagues were more positive than the course participants. This could be an indication that selection bias took place. Another explanation might be that course participants were more critical because it took effort to organize and plan the CURA-meeting, whereas their colleagues did not have to do any preparatory work.

Another limitation of this study is that respondents only used CURA once or twice when completing the questionnaires. Furthermore, they used CURA while organizational preconditions (such as support from management, having enough time to allocate to ethical reflection, etc.) were not met. Consequently, the results that are presented in this study primarily concern the feasibility of CURA. Results concerning the first perceived effects only give a first impression of the effectiveness of CURA. Therefore, further research is needed to investigate nurses’ experiences with CURA once CURA is properly implemented in their organization. This is why, following up on the outcomes of our feasibility study, we are currently conducting a three-year implementation and effectivity study on CURA in ten health care organizations (hospices, hospitals, care homes, home care) throughout The Netherlands.

## Conclusion

In this article, we discussed the feasibility and perceived outcomes of a new CES instrument called CURA. This instrument is specifically developed for nurses working in health care settings for patients with palliative care needs. Our findings indicate that the instrument itself is considered feasible and low-threshold and might help to deal with morally difficult situations in practice. The next step will be to further implement CURA in various organizational settings. During the implementation process, organizational preconditions have to be taken into account, such as commitment from all levels in the organization, a safe ethical work environment and availability of time to use CURA. We will continue to conduct research together with stakeholders on how to support nurses sufficiently in dealing with moral challenges in palliative care.

Please find CURA in the Appendix. A manual with instructions and background materials are available free of charge by sending an e-mail to: cura@amsterdamumc.nl.

## Appendix



## Data Availability

The datasets generated during and/or analyzed during the current study are available from the corresponding author on reasonable request.

## Footnotes

1 The first percentage refers to respondent group 1. The second refers to respondent group 2.
